# Optical imaging of luminescence for *in vivo *quantification of gene electrotransfer in mouse muscle and knee

**DOI:** 10.1186/1472-6750-6-16

**Published:** 2006-03-08

**Authors:** C Bloquel, C Trollet, E Pradines, J Seguin, D Scherman, MF Bureau

**Affiliations:** 1 Inserm, U640, Paris, F-75006 France; CNRS, UMR8151, Paris, F-75006 France; Université Paris Descartes, Faculté de Pharmacie, Chemical and Genetic Pharmacology Laboratory, Paris, F-75270 France; Ecole Nationale Supérieure de Chimie de Paris, Paris, F-75005, France

## Abstract

**Background:**

Optical imaging is an attractive non-invasive way to evaluate the expression of a transferred DNA, mainly thanks to its lower cost and ease of realization. In this study optical imaging was evaluated for monitoring and quantification of the mouse knee joint and tibial cranial muscle electrotransfer of a luciferase encoding plasmid. Optical imaging was applied to study the kinetics of luciferase expression in both tissues.

**Results:**

The substrate of luciferase (luciferin) was injected either intraperitonealy (*i.p.*) or *in situ *into the muscle or the knee joint. Luminescence resulting from the luciferase-luciferin reaction was measured *in vivo *with a cooled CCD camera and/or *in vitro *on tissue lysate. Maximal luminescence of the knee joint and muscle after *i.p. *(2.5 mg) or local injection of luciferin (50 μg in the knee joint, 100 μg in the muscle) were highly correlated. With the local injection procedure adopted, *in vivo *and *in vitro *luminescences measured on the same muscles significantly correlated. Luminescence measurements were reproducible and the signal level was proportional to the amount of plasmid injected. *In vivo *luciferase activity in the electrotransfered knee joint was detected for two weeks. Intramuscular electrotransfer of 0.3 or 3 μg of plasmid led to stable luciferase expression for 62 days, whereas injecting 30 μg of plasmid resulted in a drop of luminescence three weeks after electrotransfer. These decreases were partially associated with the development of an immune response.

**Conclusion:**

A particular advantage of the *i.p. *injection of substrate is a widespread distribution at luciferase production sites. We have also highlighted advantages of local injection as a more sensitive detection method with reduced substrate consumption. Besides, this route of injection is relatively free of uncontrolled parameters, such as diffusion to the target organ, crossing of biological barriers and evidencing variations in local enzymatic kinetics, probably related to the reaction medium in the targeted organ. Optical imaging was shown to be a sensitive and relevant technique to quantify variations of luciferase activity *in vivo*. Further evaluation of the effective amount of luciferase in a given tissue by *in vivo *optical imaging relies on conditions of the enzymatic reaction and light absorption and presently requires *in vitro *calibration for each targeted organ.

## Background

Methods of gene transfer to tissues are still to be optimized. Successful human gene therapy requires effective gene delivery and long term expression of the transgene. Among gene transfer methods, plasmid electrotransfer is a physical method for *in vivo *non viral gene delivery. The main target organs of this method are the skeletal muscle, which allows systemic secretion of the therapeutic protein, some tumors, as well as the skin, mainly for vaccination applications [1]. The field of applications of electrotransfer is still expanding, with the use of this method in other organs, such as cornea [2], tendon [3], liver [4], bladder [5], brain [6]. However, most of these studies focused on the establishment of transfer techniques.

To assess the feasibility, efficacy and patterns of gene transfer methods, there is a real need for simple and precise methods of evaluation. In that context, non-invasive evaluation of transfection is of great interest. This type of method allows to follow simultaneously the distribution and expression level of transferred DNA at different times reducing the number of animals used and improving statistical analysis, with each animal acting as its own control. Among different methods [7,8], optical imaging is particularly attractive because of its lower cost and easiness of realization. However, more sophisticated methods of quantitative tomography have been proposed for fluorescence [9] and are emerging for luminescence [10]. Imaging is made with a CCD (Charged Coupled Device) camera, which relies on the conversion of photons that strike a CCD pixel into spatially defined electric charges. Various reporter genes, encoding fluorescent proteins of different emission wavelength or bioluminescent proteins, are available for this method. The use of luciferase as a reporter gene requires injection of an exogenous substrate but there is no need of illumination which can perturb physiology in light sensitive tissues [11]. Luciferases are presently more sensitive than fluorescent reporters, thanks to a high quantum yield associated to a very low background level of luminescence [12,13]. Sensitivity is currently increasing with further development of brighter forms of luciferase [14] and red shifted mutants [15]. Moreover, luciferases have a rapid turnover (t_1/2 _of 3 hours) which allows real-time measurements of the protein production *in vivo *[16].

Luciferases are currently used as reporters of many biological functions [13,17]. It can be a useful tool to monitor protein-protein interactions by BRET (Bioluminescence Resonance Energy Transfer) or by LCI (Luminescence Complementation Imaging) [17]. The luciferase gene can also be used as a tracer to study tumor growth and metastasis [18,19], to follow labelled infectious bacteria or viruses [20]. Tracing of *in vivo *transgene expression after transfection of different tissues by DNA encoding luciferase is applicable [21,22]. Luciferase expression has been a useful tool to monitor the transcriptional activation of a gene [23,24], and transgenic mice can allow the evaluation of the efficacy of therapeutic interventions [25].

Consequently, if the method is to be largely used in animal research, problems of quantification remain to be studied and discussed. *In vivo*, to our knowledge only one study was presently devoted to quantification of gene transfer by optical imaging of luminescence [22]; in this work, the transfection was performed in the muscle with a luciferase-encoding AAV vector, following an *i.p. *injection of the substrate luciferin. Thus, it seemed useful to further analyze if the quantification of gene transfer by optical imaging was feasible in a simple and reliable way, possibly in several organs simultaneously, and whether a local substrate injection could be a good alternative. Our experimental model was the electrotransfer of the tibial cranial muscle [26,27] and/or the knee joint [28,29] with a plasmid encoding luciferase.

In this study, *i.p. *and local route of administration of luciferin were compared. As validated, the local injection method led to more sensitive luminescence detection than the *i.p. *substrate injection. Quantification of the *in vivo *luminescence thus obtained, was highly correlated to the *in vitro *measurements on the same organ. Reproducible measurements were obtained with the CCD camera device. This non-invasive approach to quantify gene expression in various organs was then applied to kinetic studies in the muscle and knee.

## Results

### Comparison of different routes of administration of luciferin substrate

The enzymatic reaction to visualize luciferase activity is generally obtained following intraperitoneal luciferin injection. This route of injection should allow the substrate to reach any location where luciferase is produced [21]. However the substrate local availability depends on some features such as blood perfusion and the nature of physiological barriers to cross (endothelium, extracellular matrix, epithelium ...). *I.p. *luciferin accessibility was studied in two different types of compartment: skeletal muscle and joint. We chose these two tissues in particular, since they are both interesting targets for electrotransfer, and they are very different in size, cellular type and vasculature.

**Figure 1:**
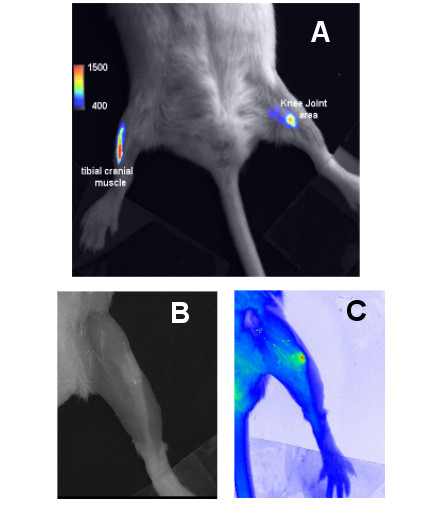
**A Luminescence of tibial cranial muscle and knee joint area after electrotransfer of a luciferase-encoding plasmid and *i.p. *injection of luciferin**. Observation was done 26 minutes after *i.p. *injection of luciferin (2.5 mg/250 μl) and 7 days after electrotransfer of 3 μg and 60 μg of luciferase encoding plasmid (pC1-luc) into the tibial cranial muscle (on the left) and the knee joint (on the right) respectively. Levels of luminescence are represented in false colours according to a scale from 400 to 1500 (whole scale 65000). 1B and 1C **Fluorescence of knee joint after injection of fluorescent albumin**. Skin of the leg was removed. Fluorescence was induced with λex = 540 +/- 20 nm and observation was done at λem > 630 nm. Panel B: At these wavelengths, without injecting fluorochrome, it was possible to see bones by transparency. Panel C Just after injection, labelled albumin is concentrated within patella of the knee joint and some diffusion of fluorescence within the joint can be observed. Levels of fluorescence are represented in false colors.

**Figure 2:**
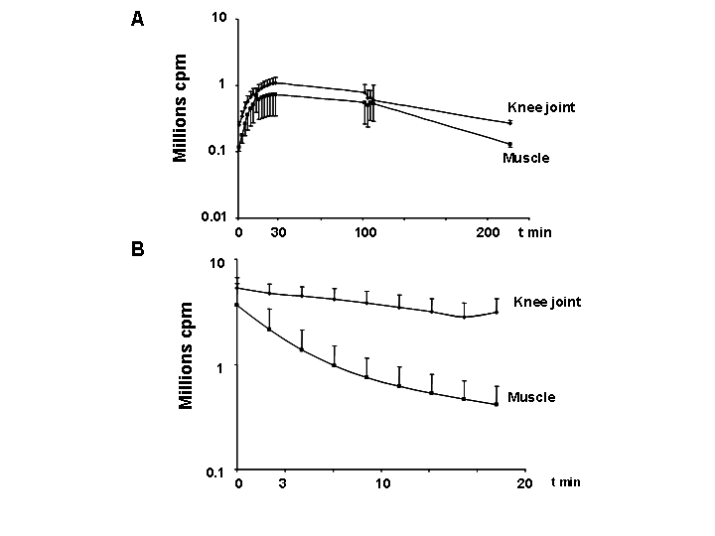
**Kinetics of luminescence of tibial cranial muscle and knee joint after electrotransfer of a luciferase encoding-plasmid and either *i.p. *or local injection of luciferin**. 3 and 60 μg of a plasmid encoding luciferase were electrotransfered into the left tibial cranial muscle and right knee joint respectively. Luminescence measurements were done 7 days after electrotransfer. Values are mean +/- SEM of the integrated values of luminescence in region of interest (ROI) of the knee joint or of the tibial cranial muscle. For all experiments, the first measurement was performed 2 minutes after luciferin injection. Panel A: *i.p. *luciferin injection (2.5 mg/250 μl), n = 5 Panel B: local luciferin injection into the muscle (100 μg/40 μl) and into the knee joint (50 μg/10 μl), n= 5/organ. Luminescence decreased with time in the muscle (t_1/2 _= 3.3 minutes), and in the knee joint (t_1/2_= 16.5 minutes).

By electrotransfering a luciferase-encoding plasmid into the right knee and the left muscle of the same mouse it was possible to observe simultaneously the luminescence in both tissues after *i.p. *injection of luciferin (Figure 1A). We controlled the procedure of injection into the knee joint using fluorescent albumin. After removal of the skin, it was possible to see better the fluorescence localization relative to bones, knee joint and muscle. Position of the knee can be seen in figure 1B. After intra-articular injection of fluorescent albumin a bright spot of fluorescence was observed at the level of the patella (Fig 1C); some diffusion of albumin was visible in the whole joint and also partly outside. From the position of the knee joint in the hind leg, it appeared that the transfection area, as shown by luminescence spot in figure 1A would be within the joint, but transfection of adjacent tissues could not be excluded. In agreement with previous results [21,22] a plateau level of luminescence was reached after about 20 to 30 min for the muscle as for the knee joint area (figure 2A). However the time required to reach the plateau level of luminescence in muscle or knee area was variable between different experiments and the amount of luciferin to inject was relatively high (2.5 mg). We thus tested if local injection of a lower amount of luciferin was a valid alternative. In addition such a procedure would more selectively evidence the knee joint transfection. We injected 100 μg/40 μl into the muscle and 50 μg/10 μl into the knee joint. Under these conditions, the substrate was in large excess relative to the expected amount of luciferase produced that should be of a few ng (in the muscle) [30]. Besides, injecting 3 to 4 times more substrate into joint or muscle did neither modify the level of luminescence nor the decrease profile, confirming that the substrate was actually in excess and thus was not a limiting factor in the targeted organ (data not shown). A fast luminescence decrease occurred as soon as the first measurements (2 minutes after local substrate injection). The t_1/2 _of the decrease was 16.5 min for knee joint and 3.3 min for skeletal muscle (Figure 2B). In addition, in both tissues a subsequent injection of the same amount of luciferin after one hour delay induced similar luminescence production (data not shown).

After electrotransfer of 3 and 60 μg of plasmid encoding luciferase into muscle and knee joint of the same mouse, respectively, the luminescence was about 4 times higher after local injection of 100 μg luciferin into muscle and 50 μg into knee than after *i.p. *injection of 2.5 mg luciferin (Table 1 – experiment 1). This 4-fold increase by the local substrate administration was confirmed by intramuscular electrotransfer of a higher plasmid dose (30 μg) as shown in table 1 – experiment 2. Nevertheless, maximal luminescence values obtained after local luciferin injection (100 μg/40 μl) were significantly correlated to those after *i.p. *injection (2.5 mg/250 μl).

We have also tested whether the sensitivity of the *i.p. *injection method could be improved when compared to the one of the muscle local procedure. For this purpose, we used an *i.p. *injection dose of 6 mg of substrate instead of 2.5 mg. The luminescence signal detected was increased, but remained below the one obtained by local injection. By *i.p. *procedure, the substrate is thus not in sufficient excess, even by injecting 6 mg of substrate, relative to enzyme concentration in the targeted tissue (Table 2).

These experiments thus highlighted the relevance of local injection with an excess of luciferin substrate, which allows luminescence detection highly correlated with *i.p. *injection. The major drawback of this method is the rapid decrease of the signal, which requires performing all measurements at the same and precisely determined time after luciferin injection. For all the following experiments, we used this local injection protocol.

### Comparison between *in vivo *and *in vitro *measurements of luciferase activity

One of the purposes of this study was to determine whether the levels of luminescence obtained from *in vivo *mouse muscle with a CCD camera were quantitatively reflective of the ones detected *in vitro *after lysis of each muscle and classical biochemical measurement with a luminometer. This part of the study was made only with muscle tissue, for which the *in vitro *biochemical assay has been optimized. Besides, measurements on the mice knee joint would be difficult both for sampling and lysis reasons. Indeed we could not accurately determine which cells were transfected in the joint, as we found expression both in the patella area but also in the whole joint region (delimited by the heads of the femur and the tibia) (Data not shown). Moreover lysis of the articular tissues would require use of collagenase which may not be compatible with the biochemical assay buffer.

To determine the conversion factor between the luminometer used for *in vitro *assays and the CCD camera used for *in vivo *imaging, we quantified the luminescence produced by different concentrations of luciferase in a 96 well plate in the presence of an excess of luciferin with both systems. Such a calibration was unfortunately not possible directly *in vivo *in mouse tissues (see Material and Methods). A good linear correlation was found between luminescence levels and luciferase amounts at different time after luciferin addition with the camera and with the luminometer, two min after substrate addition (r^2 ^= 0.994 and r^2 ^= 0.988 respectively, p < 0.001). During the first 5 minutes after luciferin addition the luminescence levels measured with the luminometer or the cooled CCD camera with the setting chosen (see Material and Methods) were very similar. Thus comparisons between measurements with both systems were made without any modifications of the values.

We determined the sensitivity of both measurement systems to detect luminescence of the luciferase-luciferin reaction in the optimized conditions *in vitro*. With the luminometer the lowest clearly detectable luminescence corresponded to a luciferase amount of about 0.01 ng (10 μl of a 1 ng/ml solution). With the CCD camera it corresponded to an amount of 0.05 ng (10 μl of a 5 ng/ml solution) due to a higher background.

Three doses of plasmid pC1luc (0.3, 3 and 30 μg) were then used to establish the comparison between *in vivo *and *in vitro *measurements. This comparison was made 8 days after i.m electrotransfer, when a maximum expression level was reached.

**Figure 3:**
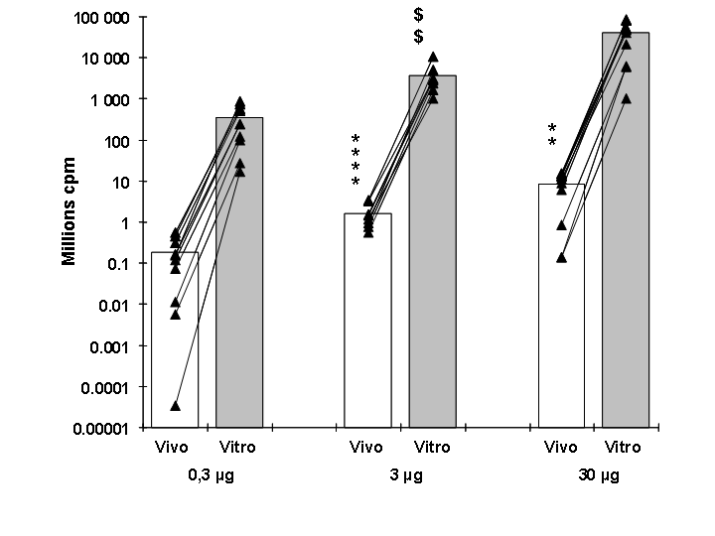
**Comparison between the *in vivo *and *in vitro *measurements of luciferase activity of tibial cranial muscle after electrotransfer with a luciferase-encoding plasmid**. Values are mean +/- SEM (n = 10) of the integrated values of luminescence in ROI of the tibial cranial muscle 8 days after electrotransfer of 0.3, 3 or 30 μg of pC1 luc plasmid as measured *in vivo *(white columns) and *in vitro *3 hours later or more (grey columns) on the same muscles. Individual values are represented by black triangles and the lines link values measured *in vivo *and *in vitro *for the same muscles. *In vivo *background luminescence was subtracted from the luminescence measured. *In vitro*, background luminescence was negligible. Dose effect: – in vitro **** p < 0.0001 between electrotransfer with 0.3 and 3 μg of plasmid ** p < 0.01 between 3 and 30 μg of plasmid – *in vivo *$$ p < 0.01 between electrotransfer with 0.3 and 3 μg of plasmid.

Figure 3 shows the luminescence levels as measured *in vivo *with the CCD camera and *in vitro *on lysates of the same muscle using the luminometer on white plates. The mean level of luminescence was higher *in vitro *than *in vivo *by 3 orders of magnitude. In spite of this difference *in vitro *and *in vivo *values of luminescence were significantly correlated (r^2 ^= 0.796 p < 0.0001; Number of paired values = 28).

**Figure 4:**
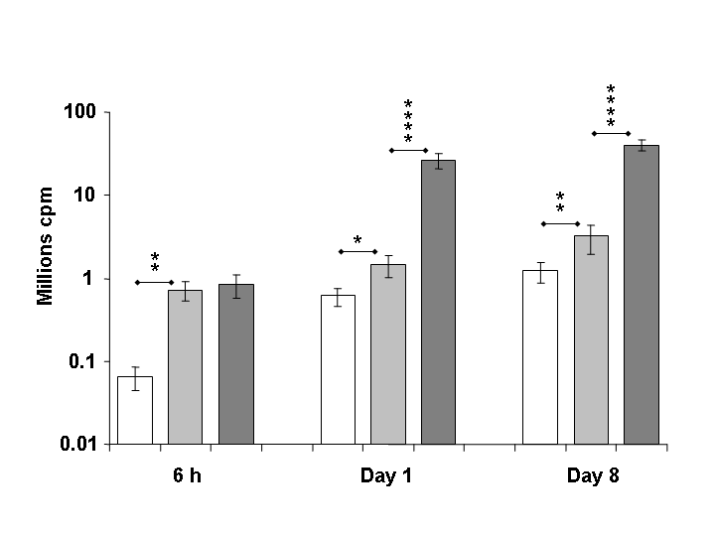
**Sensitivity of *in vivo *luminescence measurements and dose effect**. Values are mean +/- SEM of the integrated values of luminescence in ROI of the tibial cranial muscle 6 h (n = 6 to 10), 1 day and 8 days (n = 10 to 20) after electrotransfer of 0.3 μg (white columns), 3 μg (clear grey columns) and 30 μg (dark grey columns) of pCMV-luc+ into the tibial cranial muscle. For all points, background luminescence was subtracted. Statistical significance of the differences **** p < 0.0001, ** p < 0.01, * p < 0.05.

**Figure 5:**
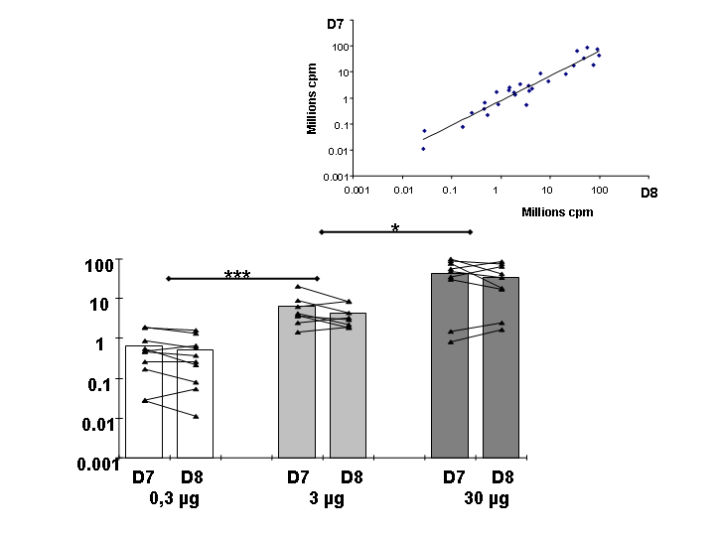
**Reproducibility of *in vivo *luminescence measurements on muscles after electrotransfer of a luciferase-encoding plasmid**. Values are mean +/- SEM (n = 8 to 10) of the integrated values of luminescence in region of interest (ROI) of the tibial cranial muscle 7 days and 8 days after electrotransfer with 0.3 μg (white columns), 3 μg (clear grey columns) and 30 μg (dark grey columns) of pC1-luc into the tibial cranial muscle. Individual values are represented by black triangles and lines link values for the same muscles at day 7 and 8 after electrotransfer. For each amount of plasmid electrotransferred, luminescence values at day 7 (D7) and 8 (D8) did not differ. For both time of measurement (D7 and D8) a significant dose effect was observed. *** p < 0.001 between 0.3 and 3 μg (for groups D7 or D8). * p < 0.05 between 3 and 30 μg (for group D7 or D8). For all points, background luminescence was subtracted Inset: Linear correlation between values of luminescence measured at day 7 and 8 after electrotransfer. R^2 ^= 0.92, p < 0.0001.

Accordingly, dose dependence between luciferase activity and injected plasmid doses was both observed *in vitro *and *in vivo *(figures 3, 4, 5).

Figure 4 shows that luminescence resulting from the reaction between luciferase and luciferin in the muscle can be detected by optical imaging as soon as 6 hours after electrotransfer of 0.3 μg of pCMV-luc+. By comparing *in vivo *and *in vitro *measurements of muscle luminescence and using *in vitro *calibration curves with recombinant luciferase, we could estimate the amount of luciferase in these muscles which was of about 3 ng (see appendix). The CCD device thus appeared as a sensible tool to quantify luciferase expression in a living organism.

### Reproducibility of *in vivo *luminescence measurements in the muscle tissue

It is known that gene expression following intramuscular electrotransfer reaches a stable maximum value one week after the injection. At this point, individual values should be consistently reproducible. We used this well known property of the electrotransfer method to validate the reproducibility of measurements with the CCD device. Mean levels of *in vivo *luminescence at days 7 and 8 after electrotransfer of 0.3, 3 or 30 μg of the pC1-luc plasmid were comparable as expected (Figure 5) and were significantly correlated (inset) (r^2 ^= 0.92 p < 0.0001). Significant correlations between values at days 7 and 8 were also observed by analyzing separately the three groups of values corresponding to the three amounts of plasmid electrotransfered (0.3 μg, r^2 ^= 0.89, p < 0.0001; 3 μg, r^2 ^= 0.59, p = 0.026; 30 μg, r^2 ^= 0.77, p = 0.0008).

### Kinetic of luciferase expression in the electrotransfered knee joint and skeletal muscle

From the above results it appears that *in vivo *luciferase quantification is a reliable methodology. We thus used this non-invasive method to study kinetics profile of luciferase activity in knee joint and skeletal muscle.

**Figure 6:**
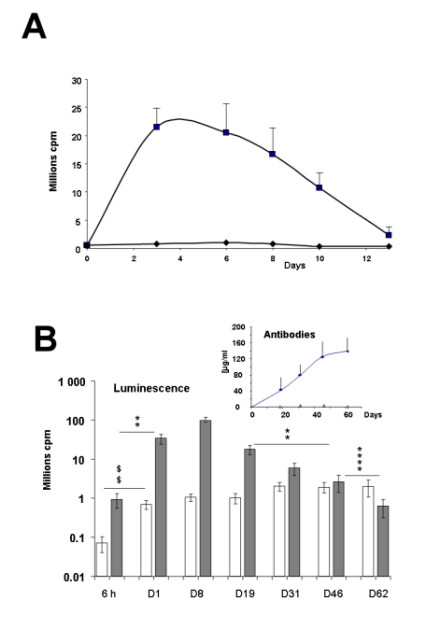
***In vivo *kinetic of luciferase expression in the knee joint and in the tibial cranial muscle and antibody concentration in blood serum after electrotransfer of luciferase-encoding plasmid**. Panel A: Mean + SEM (n = 8) of the integrated values of luminescence in region of interest (ROI) of the knee joint at different times after electrotransfer of 60 μg/10 μl of pC1-luc plasmid (black square symbols) or empty plasmid (black diamond symbols). The *in vivo *luminescence was assayed 2 minutes after luciferin injection into the joint. Panel B: Mean ± SEM (n= 6 to 10) of the integrated values of luminescence in region of interest (ROI) of the tibial cranial muscle at different times after electrotransfer of 0.3 μg (White columns) or 30 μg (grey columns) of pC1 luc plasmid. The *in vivo *luminescence was assayed 2 minutes after luciferin injection into the electrotransfered muscle. For all point background luminescence was subtracted. Statistical significance for comparisons of luminescence between different times after electrotransfer of 30 μg of plasmid **** p < 0.0001, ** p < 0.01; after electrotransfer of 0.3 μg of plasmid $$ p < 0.01 Inset: Mean + SEM (n = 5) of the concentration of antibodies against luciferase in the blood serum at different times after electrotransfer of 0.3 μg (empty triangles) or 30 μg (black diamon) of pC1 luc plasmid into the muscle.

The expression of luciferase in the knee joint area was followed for two weeks after electrotransfer of 60 μg of pC1-luc or the same dose of a control plasmid with no encoding sequences. A maximum value of luminescence was reached between 3 and 6 days after electrotransfer. Expression of luciferase gradually decreased thereafter and returned close to the control level 13 days after the electrotransfer (Figure 6A).

In skeletal muscle, luciferase expression was stable for at least two months following the electrotransfer of either 0.3 μg plasmid (Figure 6B) or of 3 μg (data not shown). By contrast, a decrease of luminescence at day 19 for the high dose of 30 μg plasmid, was observed. At day 62 the luminescence for muscle electrotransfered with 30 μg plasmid was comparable to that of muscle electrotransfered with 0.3 μg plasmid and was significantly decreased relative to that at 19 days.

Thirteen days after electrotransfer of knee with 60 μg plasmid encoding luciferase, the presence of antibodies against luciferase was detected in the serum of treated mice (65 ± 17 μg/ml). No antibodies were detected in serum of mice until 61 days after electrotransfer of tibial cranial muscles with 0.3 μg of plasmid. By contrast, when the amount of plasmid injected in the muscles was 30 μg, antibodies were clearly detected in serum at an increasing level between days 19 to 61 after electrotransfer (inset Figure 6B).

## Discussion

The use of luciferase as a reporter gene is a powerful tool for *in vivo *optical imaging of the transfection of various organs [21]. Until now, luminescence has been mainly induced by substrate (luciferin) injected intraperitoneally [21,22]. We have shown that this approach allows simultaneous visualization of the transfection of tibial cranial muscle and knee joint area in the same mouse. This indicates that the substrate can reach even a small and less perfused compartment such as the knee joint. Such a detection method is of particular interest for the knee, since quantitative *in vitro *measurement of luciferase activity in this tissue is difficult and has been reported only for bigger animals such as the rat [28,29]. Following luciferin *i.p. *injection, curves of luminescence versus time had a similar pattern in both muscle and joint. However, the time to reach maximum level of expression was variable from one experiment to the other, and the amount of luciferin used was relatively high. Moreover, by using a higher dose of substrate (6 mg), we demonstrated that the maximal level of luminescence is not reached with the commonly used dose of 2.5 mg substrate. In addition, selective transfection of the knee joint is difficult and sometimes neighboring tissues can also be transfected. We therefore tested if the local injection of lower amounts of luciferin could be used alternatively, the other advantage being a more selective estimation of transfection for the knee joint.

### Comparison between *i.p. *and local luciferin administration

By local injection of the substrate, a maximal value of luminescence was detected in both muscle and joint as soon as the first CCD camera observation. Luminescence then decreased rapidly in the muscle and somewhat slower in the knee joint. Most importantly, luciferin local injection led to a four fold luminescence increase over 2.5 mg luciferin *i.p. *injection. Additionally, *i.p. *injection of a higher amount of 6 mg luciferin still did not allow to reach the luminescence level obtained after local injection of 100 μg luciferin. Maximal luminescence levels obtained after *i.p. *(2.5 mg) or local (0.1 mg) injection of luciferin were highly correlated in both knee and muscle, even when different amounts of plasmid were administrated in the muscle. This indicates that both procedures of luciferin injection can be used for monitoring variations of luciferase activity *in vivo *independently of the tissue concerned or of the amount of plasmid electrotransfered. Local procedure allows reduced substrate consumption and is more sensitive since it induces more luminescence. This improved sensitivity after local injection of luciferin was also shown by Li *et al *who detected luciferase activity of transfected rat muscles for a longer period after local than after *i.p. *injection of luciferin [31]. Nevertheless intraperitoneal injection of high amounts of luciferin remains a useful technique when the site of luciferase production is difficult to reach or is unknown. In addition, the time to reach peak level of luminescence in one tissue after *i.p. *luciferin injection is related to the time for the substrate to access its target. This might, for example, provide information on blood perfusion.

We observed a slower luminescence decrease after local injection of luciferin in the knee joint than in the muscle. This decrease of luminescence with time *in vivo *could be related to a decrease of substrate concentration because of diffusion and washout by the circulation. Such an hypothesis is sustained by the stability of luminescence on cells *in vivo *[32]. However, we used a large excess of substrate (100 μg luciferin vs < 100 ng luciferase in the muscle i.e at minimum 1000 times more substrate), and can assume that diffusion will have not a great effect. Besides, increasing the substrate concentration did neither modified significantly the maximum value of luminescence nor the kinetic of luminescence decrease, highlighting no effect of the substrate concentration on the signal. Lastly addition of luciferin (100 μg/40 μl) to isolated electrotransfered tibial cranial muscle in a multiwell plate induced the same type of decreased kinetics, despite a large excess of substrate and the absence of washout problems. These differences also cannot be due to kinetic of diffusion of the luciferin inside cells since a similar kinetic profile is obtained by mixing recombinant luciferase to luciferin in the buffer used for *in vitro *measurements. Moreover the amount of luciferase protein in the cells seemed to remain constant and the cofactors required for the reaction (ATP, O_2_, Mg^2+ ^principally) were in sufficient excess since a new addition of substrate after return to luminescence background induced a similar luminescence level in both muscle and knee joint. In conclusion, decrease of luminescence with time seems essentially related to the reaction media and/or to enzyme complexation.

Kinetic differences between muscle and knee might thus be linked to the reaction media which can differ according to the tissue. For example it has been reported that the kinetic of light production is related to the concentration ratio between the enzyme and ATP [33]. The reaction can also lead to the production of inhibitors of the luminescence [34]. Lastly some molecules present in cells stimulate [34,35] and/or inhibit the luminescence [34,36].

As a consequence, luciferase quantification in different tissues is sensitive to the conditions of the reaction. In a given tissue the quantification will rely on the hypothesis that these conditions of reaction are the same throughout the study.

### Validation of optical imaging as a tool to quantify luciferase activity *in vivo*

Until now, optical imaging with luciferase was mostly presented as a semi quantitative technique. From the above consideration it was thus essential to verify if the values measured by optical imaging in these experiments were consistent with values obtained by the classical luminescence quantification method: *in vitro *measurements on the supernatant of tissues homogenized in lysis buffer.

We observed a significant correlation between *in vivo *and *in vitro *measurements. This result confirms the results obtained by Wu *et al *[22], but with a local injection of substrate, and thus a more sensitive method, and for a wide range of plasmid dose. In addition, a clear dose effect was observed *in vivo *8 days after intramuscular electrotransfer of 0.3, 3 or 30 μg of plasmid, which is consistent with *in vitro *measurements of this study and confirmed the *in vitro *results of a previous work [37]. Moreover, optical imaging system was sensitive enough to detect luminescence resulting from the reaction of luciferase with luciferin as soon as 6 hours after intramuscular electrotransfer of 0.3 μg of pCMV-luc+ plasmid. Finally a good reproducibility of luminescence measurements was observed at times for which transfection level of the muscle is known to be stable [30].

We can thus assume that variations of luminescence detected with a cooled CCD camera are sensitive enough, accurate and well related to the effective amount of luciferase present in the studied tissue.

Taking into account differences between measurement systems (conversion factor between the two devices, see Material and Methods), our data highlight that luminescence *in vivo *was largely lower when compared to that *in vitro*. Understanding this discrepancy is necessary to improve sensitivity of *in vivo *measurements. One factor could be the absorption and scattering of light by the tissue. But luminescence attenuation is not sufficient through a few millimeters of tissue [22,38]. The main cause of observation of a lower luminescence *in vivo *must be linked to the difference in reaction media for *in vivo *and *in vitro *measurements. Indeed media used *in vitro *are optimized in order to maximally enhance the luminescence of the luciferase-luciferin reaction. We thus observed that when luciferase was diluted in Tris buffer (pH 8) the luminescence produced after addition of the substrate and ATP was about 2 orders of magnitude lower than when diluted in lysis buffer (which also contain Tris). We verified that part of this improvement was due to the presence of dithiothreitol (DTT) known to avoid enzyme degradation [35] and to the triton detergent, both present in the lysis buffer (data not shown). In addition, *in vitro *measurements were usually performed in multi-well white plates, in which the light emission measured was higher by one order of magnitude than the luminescence level measured in black plates (data not shown), as expected from previous studies [39]. Taking into account these two factors (difference of reaction media and the type of plates used) seems sufficient to explain the difference between *in vivo *and *in vitro *measurements. Nevertheless, only tomographic measurements could be appropriate to collect the whole luminescence emerging from the muscle. Indeed, fluorescent tomographic measurements were proven more sensitive than planar measurements [9].

Our results highlight that luminescence quantification in one tissue is rather accurate and can be related to the effective amount of luciferase but requires an additional calibration step to put in adequacy *in vivo *to *in vitro *measurements. *In vivo *luminescence levels in different tissues are not directly related to their luciferase contents because of differences in the condition of *in vivo *enzymatic reaction and in tissue absorption depending on the location of the tissues. Thus absolute quantification in studies implying several tissues require preliminary calibration for all the tissues involved.

### Kinetics of luciferase expression in the muscle and the knee

We have studied kinetic profiles of luciferase activity in the muscle or in the knee joint electrotransfer of a luciferase-encoding plasmid. To our knowledge electrotransfer into the knee joint was only reported for rats in two studies [28,29] and non-invasive detection of luciferase activity in the electrotransferred mouse knee joint has never been done previously.

High and long term (> 2 months) level of luciferase expression after intramuscular electrotransfer has been well documented. Here, we confirm by optical imaging the stability of luciferase expression for at least 62 days in tibial cranial muscle after electrotransfer of 0.3 or 3 μg of luciferase-encoding plasmid. In contrast, a decrease of luciferase activity occurred three weeks after electrotransfer of the higher dose of 30 μg of plasmid, concomitantly to the production of antibodies against luciferase. Similar immune responses were also observed by others, after pC1luc plasmid DNA transfer into the liver of C57Bl6 mouse [40]. After electrotransfer of 60 μg of plasmid into the mouse knee joint, luciferase activity reached a peak between 3 to 6 days and returned to the control level two weeks after electrotransfer. A similar kinetic profile was also observed by Ohashi *et al *by *in vitro *evaluation of the transgene expression in the synovium [29]. In contrast Grossin *et al *observed expression of an electrotransfered plasmid encoding for GFP for a longer period and localized mainly in the knee patellae [28]. These differences may be related to the use of different electrotransfer conditions (injection site, electric conditions and electrodes). In the present study, by using optical imaging, we have detected the luminescence of the whole knee and it was not possible to identify the particular type of cells transfected under the specified conditions. Decrease of luciferase activity in this tissue can be partially explained by synovial cell turnover. Indeed, antibodies against luciferase were detected as soon as 13 days after electrotransfer of a high dose of plasmid. In muscle, the decrease of luciferase activity and the concomitant antibodies detection was only observed while injecting a higher dose of plasmid. These results confirm that a non-self protein expressed into myofibres seems to be not detected by the immune system when its concentration is below a threshold value as demonstrated by Miller *et al *[41]. With higher dose of electrotransfered plasmid, the observed immune response can occur via multiple mechanisms [42]. It is known that electrotransfer enhances the specific immune response both by increasing transgene expression and by inducing inflammation [43,44] which is more important when high amounts of plasmid are injected [45,46] but is reversed two weeks after electrotransfer. In summary we observed a specific humoral immune response against luciferase and probably cytotoxicity against muscular fibres expressing the luciferase resulting in decrease of the luciferase activity. These two types of specific immune reactions have been observed in several studies in DNA vaccination models (see for example [42]).

## Conclusion

In conclusion we have shown here that optical imaging allows *in vivo *visualization and quantitative assay of luciferase activity in two different types of tissue in the mouse model, the muscle and the knee joint, which defines a smaller and less vascularized compartment. This observation can be made after *i.p. *or local injection of the luciferin substrate. We showed that local injection allowed a more sensitive detection of luciferase activity with lower substrate consumption. Luminescence observed in such conditions can be assumed to be independent from uncontrolled parameters as diffusion of the substrate to the target cells or circulation washout.

In the field of gene therapy, this detection method could be useful for promoter efficacy studies and optimization of gene transfer methods, in muscle, joint or other tissues accessible to the substrate of luciferase. Alternatively, luciferase can be used as a tracer by its fusion with other transgenic proteins of interest that cannot be easily quantified and localized, or by transfecting cells of interest with a luciferase encoding plasmid. This might greatly enhance the scope and further applications of the present study. However, care must be taken in future studies toward the development of a possible immune response against the reporter transgenic protein when high transgene expression levels are required.

## Methods

### Animals

*In vivo *studies were done using 6 to 8 weeks old female C57Bl/6 or Swiss mice (Charles River, L'Arbresle, France). Prior to all procedures, the animals were anaesthetised by intraperitoneal injection of ketamine and xylazine (Bayer Pharma, Puteaux, France) (100 and 10 mg/kg, respectively). Studies were conducted following the recommendations of the European Convention for the Protection of Vertebrates Animals used for Experimentation and the local Ethic Committee on Animal Care and Experimentation.

### Plasmid DNA

Plasmid pC1-luc is an expression vector carrying a modified cytosolic firefly luciferase gene (luc+) cloned downstream of a CMV promoter.

In one experimental series (Figure 4) we used plasmid pXL 3031 (pCMV-luc+) which also carries the modified cytosolic firefly luciferase gene (luc+) under the control of a CMV promoter (gift from Aventis Pharma [47]); it leads to comparable levels of expression of the luciferase.

Plasmid DNA was purified using Endo-free Qiagen Maxiprep kits (Qiagen, Germany). All dilutions were done in saline (NaCl 0.9 %).

### Injection and electrotransfer of plasmid into the tibial cranial muscle and the knee joint

Plasmid DNA (0.3 to 30 μg/30 μl) was injected into the tibial cranial muscle. Twenty seconds later, 8 square-wave electric pulses (200 V/cm, 20 ms, 2 Hz) were delivered through two stainless steel plate electrodes placed each side of the shaved leg, using an ECM 830 BTX electropulsator (Genetronics, San Diego, CA, USA) [26,30].

For electrotransfer of plasmid into the knee joint, 60 μg (in 10 μl) were injected into the joint by using a hamilton syringe. Twenty seconds after the injection, 12 electric pulses of 250 V/cm, 20 ms each at 2 Hz (personal communication of Florence Apparailly) were applied using a pair of stainless steel parallel electrodes (1 cm long, 0.3 cm large, and 4.5 mm apart) placed at each side of the knee (anterior-posterior direction).

For all experiments electrical contact with the skin was ensured by application of a conductive gel.

### Optical imaging of luminescence

Luciferin diluted in PBS (Promega, Madison WI, USA) was injected intraperitonealy (*i.p.*, 2.5 mg/250 μl) or locally into the tibial cranial muscle (100 to 400 μg/40μl) or the knee joint (50 to 150 μg/10 μl). Optical imaging was performed with a cooled CCD camera (Apogee, Ap47p, Auburn Calif, USA) placed in a black box and equipped with a 60 mm lens opening at F 2.8 (Nikon, Japan). Distance from the lens to the mouse was of 30 cm. Operating temperature was set at -25°C. Duration of luminescence acquisition was between 30 s and 240 s and was initiated 2 minutes after injection of the substrate. Quantum efficiency is > 90 % in the λ range of 500 to 680 nm. Luminescence levels were integrated in region of interest (ROI) drawn by hand around luminescence zones corresponding to the tibial cranial muscle and/or knee joint area as estimated from superposed optical image of the mice. Background luminescence was subtracted according to values obtained in ROI drawn on a non transfected zone of the mice (software β Vision+ from Biospace Mesure, Paris, France). ROI were very similar from one experiment to the other. When following kinetic of the luciferase-luciferin reaction strictly the same ROI were used for luminescence images taken at different times. In some experiments, when the luminescence signal reached background level, mice were euthanazied to remove tibial cranial muscles which were then frozen at -20°C for further *in vitro *luciferase assays.

### Optical imaging of fluorescence

To asses the localization of injected plasmid into the knee joint, fluorescent albumin (BSA, Alexa Fluor 594 conjugate from Molecular Probe, Oregon, USA) was injected into this tissue as described in the previous section. Fluorescence was induced with λex of 540 +/- 20 nm and observed at λem > 630 nm. Lens aperture: F = 11 and acquisition time: 0.05 sec

### Biochemical luciferase activity assays

After sample thawing, each muscle was homogenized in 1 ml of cell culture lysis reagent (Promega, Charbonnière, France) supplemented with protease inhibitor cocktail (Boehringer Mannheim, Mannheim, Germany) (one tablet for 50 ml). After centrifugation at 12,000 rpm for 10 min at 4°C, the luciferase activity on 10 μl of the supernatant was assessed in 96 wells white plate using a Wallac Victor luminometer (EG&G Wallac, Evry, France), by integration of the light produced during 1 s, starting after the addition of 50 μl of luciferase assay substrate (Promega) to the lysate. Results were given for the whole muscle in counts per second (cps) for 1 ml of supernatant. It was not necessary to substract background luminescence, which was very low (less than 50 cps).

### Determination of a conversion factor for comparison of *in vivo *and *in vitro *measurements of luminescence

Different amounts of luciferase (0.001 to 10 nmoles) in 10 μl were distributed in wells of a black 96 wells plate, and 50 μl of luciferin solution (Promega, Madison WI, USA) were then added in each well. Measurements with the luminometer or the cooled CCD camera were initiated 2 minutes after addition of the substrate. Conversion factor is the ratio of measured values with both systems at a given time.

It was not possible to establish directly a calibration curve *in vivo *after injection of different amounts of luciferase into the knee joint or the muscle. Indeed injected luciferase is distributed in the extracellular space and the addition of luciferin does not induce detectable luminescence essentially because of the lack of ATP. We verified that addition of ATP with luciferin allowed to obtain luminescence but it is not obvious that in these conditions the luminescence measured would be equivalent to that measured for the same amount of intracellular luciferase.

### Titration of antibodies against luciferase in serum

At different times after electrotransfer, blood samples were collected by retro orbital puncture of anaesthetized mice, centrifuged at 3000 rpm for 10 minutes at 4°C, and serums were conserved at -80°C for further ELISA antibody detection. Microtiter plates were coated overnight at room temperature with luciferase (1 μg/ml, Promega, Madison WI, USA) in 100 μl of PBS. The wells were then saturated for 30 minute at 37°C with 100 μl of blocking buffer (PBS/0.1% Tween 20/0.2% gelatine). Serum were diluted at 1:1000 in 100 μl of blocking buffer and incubated for 1 h at 37°C. Then 100 μl of horseradish peroxidase-conjugated goat anti-mouse secondary antibody (diluted 1:2000) in wash buffer (PBS/0.1% Tween 20) was added to the plates and incubated for an additional hour at 37°C. Bound antibody was detected colorimetrically by using ortho-phenilenediamine (Sigma, USA) as a substrate, diluted in sodium citrate buffer (0.1 M sodium citrate plus 0.1 M citric acid, pH 5.0) to a final concentration of 1 mg/ml. Color was assayed at 492 nm after 5 min incubation at room temperature. Serum samples from individual animals were assayed in triplicate. As a control and for quantification, a serial dilution of a monoclonal antibody anti-luciferase (Sigma, L2164) was used ranging from 0 to 2 μg/ml in blocking buffer.

### Statistics

Variance analyses on log values of the measured parameters, and a protected least significance test of Fisher for comparison between treatments have been used. Correlations between groups of paired values were established by a linear regression analysis. Values of several separate experiments have been gathered when variance analysis showed no significant difference between these experiments.

## Authors' contributions

CB performed electrotransfer and optical imaging of the knee joint. CT performed antibody detection and contributed to experiments on muscles. Technical and intellectual contribution of CB and CT were equivalent and they both contributed highly to criticism and writing of the manuscript. EP was more particularly involved in experiments of comparisons between *in vitro *and *in vivo *measurements of luciferase activity. JS participated to experiments of electrotransfer and optical imaging of the muscle and data analysis. DS is director of the research unit and helped to draft the manuscript. MFB was responsible for the design and development of this study and writing of the manuscript. He performed a great part of electrotransfer and optical imaging experiments and all statistical analyses.

## Appendix

### Estimation of luciferase amount in the tibial cranial muscle from *in vivo *luminescence measurement

It is not possible to evaluate exactly the amount of luciferase corresponding to *in vivo *detected luminescence. But estimation is possible. Indeed from linear calibration curve y = Ax + B, we know the relation between luminescence *in vitro *y and the amount of luciferase x. Next from experiment in figure 3 we know the mean ratio k between luminescence measured *in vitro *y and luminescence *in vivo *y' for the same amount of luciferase i.e k = y/y'. It results that amount of luciferase *in vivo *x' ≈ (y'.k - B)/A. Hypothesis being that the ratio between luminescence *in vitro *and *in vivo *is constant and not dependant of the amount of luciferase. Results of figure 3 indicate the level of approximation of such a hypothesis. In addition such a calculation cannot be extrapolated to other tissues where the luminescence reaction is different.

## Figures and Tables

**Table 1:** Comparison between local and *i.p. *injection of luciferin to induce luminescence after electrotransfer of a plasmid encoding luciferase into the knee joint or tibial cranial muscle

	**Experiment 1 (n = 5)**				**Experiment 2 (n = 8)**	
	**Knee 60 μg**		**Muscle 3 μg**		**Muscle 30 μg**	
	
	***Local***	***i.p.***	***Local***	***i.p.***	***Local***	***i.p.***
**Luminescence **Millions units/min	4.37 ± 1.40	1.18 ± 0.25	3.08 ± 1.81	0.74 ± 0.38	20.41 ± 5.62	4.86 ± 1.20
**Ratio local/i.p.**	3.46 ± 0.76		3.55 ± 0.96		3.91 ± 0.38	
**Correlation**	r^2 ^= 0.817		r^2 ^= 0.929		r^2 ^= 0.980	
**local/i.p.**	*p = 0.0351*		*p = 0.0082*		*p < 0.0001*	

Values are mean ± SEM of the integrated values of luminescence in ROI of the muscle or of the knee. In the first experiment, 60 and 3 μg of luciferase-encoding plasmid were electrotransferred into one knee joint and one tibial cranial muscle respectively. Luminescence was measured with the CCD camera 3 days after electrotransfer. In the second experiment, 30 μg of plasmid were electrotransferred into both tibial cranial muscles and luminescence was measured 7 days later. For all the experiments, luciferin was injected either locally (50 μg in the knee, 100 μg in the muscle) or *i.p. *(2.5 mg).

**Table 2:** Comparison of luminescence of tibial cranial muscles after electrotransfer of a luciferase-encoding plasmid and injection of luciferin i.p. (2.5 mg or 6 mg) or locally (0.1 mg)

**Luciferin administration**	***i.p.***		***local***
	***2.5 mg***	***6 mg***	***0.1 mg***
**Luminescence **10^5 ^units/min	2.05 ± 0.32	7.06 ± 2.79	18.42 ± 2.68

Values are mean ± SEM (n = 6 to 12) of the integrated values of luminescence in ROI of the tibial cranial muscle 3 days after electrotransfer of 3 μg of pC1 luc plasmid as measured *in vivo *after luciferin injection *i.p. *(2.5 mg/250 μl, n = 6 or 6 mg/250 μl, n = 6) or locally (0.1 mg/40 μl n = 12). Local injection of luciferin was performed 2 hours or more after the *i.p. *injection. Background luminescence was subtracted from the luminescence measured.
